# Draft Genome Sequence of *Nocardia jinanensis*, an Opportunistic Bacterial Pathogen That Causes Cellulitis

**DOI:** 10.1128/genomeA.00593-16

**Published:** 2016-07-21

**Authors:** Alolika Chakrabortti, Jinming Li, Zhao-Xun Liang

**Affiliations:** a School of Biological Sciences, Nanyang Technological University, Singapore; b Department of Bioinformatics, School of Basic Medical Sciences, Southern Medical University, Guangzhou, Guangdong, China

## Abstract

The draft genome sequence of *Nocardia jinanensis*, an opportunistic pathogen that can cause skin infections, reveals genes that may contribute to the lifestyle and pathogenicity of *N. jinanensis*. The genome also reveals the biosynthetic capacity of *N. jinanensis* in producing mycolic acids, siderophores, and other polyketide and nonribosomal peptide-derived secondary metabolites.

## GENOME ANNOUNCEMENT

*Nocardia* is a genus of rare actinomycetes that is partially acid-fast in nature and characterized by a microscopic appearance of branching hyphae (1). Various *Nocardia* strains have been isolated from aquatic and terrestrial habitats as well as the tissue of infected patients. *Nocardia* are capable of producing secondary metabolites, some of which are likely to contribute to the pathogenicity of pathogenic *Nocardia* strains. The *Nocardia jinanensis* strain NBRC 108249 (CGMCC 4.3508, DSM 45048) was isolated from soil samples and is considered an opportunistic pathogenic strain that causes a form of skin infection called cellulitis (2). The pathogenesis of *N. jinanensis* seems to differ from that of the pathogenic *Nocardia* strains *N. farcinica* and *N. brasiliensis*. We sequenced the genome of *N. jinanensis* to gain a better understanding of the pathogenicity of *N. jinanensis* and its biosynthetic capacity.

The genomic DNA of *N. jinanensis* was isolated and purified using the TIANamp bacteria DNA kit. Genome sequencing was performed using a whole-genome shotgun technique (HiSeq Illumina platform), and the DNA fragments were assembled using SOAP*denovo* by Macrogen, Inc. (South Korea). The draft genome of *N. jinanensis* has a G+C content of 67.3% and a size of 5.04 Mb. The genome seems to be relatively small compared to the genomes of *N. farcinica* (6.3 Mb), *N. cyriacigeorgica* (6.2 Mb), and *N. brasiliensis* (9.4 Mb). Phylogenetic analysis of *Nocardia* spp. based on 16S RNA sequences suggests that *N. jinanensis* belongs to the same clade as the pathogenic *N. farcinica* and *N. cyriacigeorgica* in the phylogenetic tree consisting of 78 *Nocardia* species. The genome of *N. jinanensis* contains the orthologs of a large number of putative pathogenic genes of *N. farcinica* (3), such as the Mce and YbrE virulence factors used for mammalian cell invasion and infection, superoxide dismutases, antigenic proteins/transporters, esterases, and hemolysin. The presence of the putative pathogenic genes provides support for the lifestyle and pathogenicity of *N. jinanensis* as an opportunistic pathogen.

Mining of biosynthetic gene clusters using antiSMASH 3.0 (4) suggests that the genome contains more than 20 biosynthetic gene clusters that encode polyketide synthase (PKS), nonribosomal peptide synthase (NRPS), terpene cyclase, glycosyltransferases, and other enzymes. The genome contains several unique biosynthetic gene clusters that indicate that *N. jinanensis* can produce secondary metabolites that are unique to the strain. We identified eight NRPS gene clusters and two PKS gene clusters in the genome. One of the PKS gene clusters shares high similarity with the mycolic acid gene cluster from *Mycobacterium tuberculosis*. This cluster is likely to be involved in the biosynthesis of mycolic acids, considering that it contains all the genes required for mycolic acid biosynthesis, including the genes that code for the iterative PKS, AMP-dependent ligase, carboxyl transferase, and other essential enzymes (5). The genome also contains two PKS-NRPS hybrid gene clusters. One of the PKS-NRPS gene clusters bears resemblance to the nocobactin cluster of *N. farcinica* and is also conserved in *N. cyriacigeorgica* GUH-2 (6).

### Nucleotide sequence accession numbers.

The genome sequence can be found in GenBank with the accession numbers LNDA01000001 to LNDA01000107.
